# Tracking demands for seeking psychological help before and during the COVID-19 pandemic: a quanti-qualitative study

**DOI:** 10.1186/s41155-023-00264-0

**Published:** 2023-08-29

**Authors:** Bruna M. C. Coutinho, Luis F. C. Anunciação, Jesus Landeira-Fernandez, Thomas E. Krahe

**Affiliations:** Department of Psychology, Pontifical Catholic University of Rio de Janeiro, Rua Marquês de São Vicente 225 Gávea, Rio de Janeiro, RJ CEP: 22451-900 Brazil

**Keywords:** Coronavirus, SARS-CoV-2, Mental health, IRaMuTeQ, Therapy, Complaint

## Abstract

The COVID-19 pandemic has placed unprecedented burdens on individuals and communities around the world. The isolation, fear, and uncertainty caused by the virus has led to increased rates of anxiety, depression, and other mental health issues. The pandemic has also had a disproportionate impact on individuals and communities with low income and socioeconomic status.

**Objective**

To shed light on the consequences of the pandemic on individuals from minorities and low-income areas, we investigate the main reasons that led patients who were referred to a social clinic of a private university in Rio de Janeiro to seek psychological treatment before (2019) and during the pandemic (2020 and 2021).

**Methods**

We conducted a quanti-qualitative study with a lexical analysis that evaluated 549 complaint forms of patients seeking treatment in these two distinct periods. Our analyses included descending hierarchical analysis (DHA) and correspondence factor analysis (CFA).

**Results**

Family dynamics and communication factors play a dominant role in the reason for seeking therapy and psychological treatment. Additionally, our study suggested an increase in anxiety and panic attacks among other mental health issues associated with grief and losses during the pandemic years.

**Conclusion**

Based on these analyses, we can begin to identify a few changes in the main demand and redirection of complaints of patients during the period of COVID-19.

**Supplementary Information:**

The online version contains supplementary material available at 10.1186/s41155-023-00264-0.

## Introduction

The COVID-19 pandemic has had a profound and far-reaching impact on society, affecting nearly every aspect of life (Heitzman, 2020). The measures taken to slow the spread of the virus have drastically altered the life of the great majority of the population worldwide, and hence has been termed as a global health emergency (Lewis et al., 2022; Zullo et al., 2021). In particular, the increase in mental health issues related to the COVID-19 pandemic has been an issue of major concern (Penninx et al., 2022). It is known that the pandemic has affected social groups in different ways and research points to more severe mental health consequences in children (Kumar & Nayar, 2021), adolescents (Jones et al., 2021), women (Almeida et al., 2020), people living in poverty (Bassey et al., 2022), the elderly (Grolli et al., 2021), and risk groups in general (Cui et al., 2022).

The isolation that comes with lockdowns, the fear of contracting the virus, disruption of daily routine and activities, grief and trauma associated with the loss of loved ones, and limited social interactions has led to an increase in mental health issues such as anxiety and depression (Albuquerque & Santos, 2021; Goveas and Shear, 2020; Ritchie et al., 2020; Boserup et al., 2020; Bradbury-Jones & Isham, 2020; Campbell, 2020; Davide et al., 2020; Kofman & Garfin, 2020; Nicolini, 2020; Sediri et al., 2020; Volkow & Blanco, 2020).

In particular, it is known that gender plays an influence on mental health and with the effects of the COVID-19 pandemic it was no different (Sediri et al., 2020). Self-reported levels of stress, anxiety, depression, and posttraumatic stress symptoms were significantly associated with gender (Almeida et al., 2020). This may occur due to the fact that women have a higher prevalence of risk factors known to intensify the stress caused by the pandemic, which include chronic environmental strain, preexisting depressive and anxiety disorders, domestic violence, and parenting (Almeida et al., 2020; Usmani et al., 2021). In addition to that, women may also have gone through specific contexts such as pregnancy, postpartum and miscarriage, known to be of high vulnerability to mental health problems (Ahmad et al., 2021; Usmani et al., 2021). For the elderly, the pandemic’s social changes cause emotional suffering and frustration in those with mental disorders (Cui et al., 2022; Grolli et al., 2021). Isolation for prolonged periods of time can lead to feelings of loneliness and anger, and simple day-to-day activities become stressors (Grolli et al., 2021). As for the low-income population, the suffering is related to the lack of access to information, and quality health services, along with financial stress and the difficulties of staying in isolation, and in protected spaces (Bassey et al., 2022).

Moreover, studies have shown that individuals who have lost their jobs or experienced financial hardship as a result of the pandemic are at a higher risk of experiencing depression, anxiety, and stress (Bassey et al., 2022; Maital & Barzani, 2020). Evidence from recent research has also demonstrated that such mental health problems associated with the COVID-19 pandemic can persist for a long time (Meherali et al., 2021).

In addition to the health and economic impacts, the COVID-19 pandemic has also highlighted and exacerbated existing social inequalities in developing countries (Shoib & Arafat, 2021; Shoib et al., 2022). For example, vulnerable populations such as the poor, informal workers, and those living in slums have been disproportionately affected by the pandemic, as they often lack access to adequate healthcare, housing, and other basic necessities (Bassey et al., 2022; Lewis et al., 2022). Brazil is no exception to this rule (Carvalho et al., 2021). The country was considered one of the epicenters of the COVID-19 pandemic, and the health-care system was overwhelmed by the number of patients needing care (Carvalho et al., 2021; de Oliveira et al., 2022). Additionally, the pandemic has highlighted and exacerbated existing social and economic inequalities, with low-income and marginalized communities being disproportionately affected by the virus (Lewis et al., 2022; Yoshikawa et al., 2012).

Therefore, the characterization of the impact of the COVID-19 pandemic on the mental health of vulnerable populations may prove to be important for our understanding of the consequences associated with this global health problem. To shed light on this issue, we employed a quanti-qualitative approach to investigate and compare the reasons why individuals with low income and socioeconomic status sought psychological help before and during the COVID-19 pandemic. Overall, we found that family dynamics and communication factors play a dominant role in the reason for seeking therapy and psychological treatment. Also, our data suggests an increase in anxiety and panic attacks, among other mental health issues associated with grief and losses, during the pandemic years. By investigating whether the reasons for patients seeking a social clinic have changed, we generate data that can be useful to contribute to the literature and better inform health services regarding conduct, planning, risk factors, and susceptibility of cases.

## Material and methods

### Sample

A total of 549 patient’s reports, collected between March, 2019 and December, 2021 at the Service of Applied Psychology (SAP) of the Pontifical Catholic University of Rio de Janeiro (PUC-Rio), were used in this study. We opted to use patient’s mental health reports from 2019 because it is the year juxtaposed to 2020—the year that COVID-19 pandemic started in South America (González-Bustamante, 2021; Sakellariou et al., 2020). It is our understanding that by shortening the time gap between the “before” and “onset” periods of the pandemic, we minimize social-economic differences and other demographic factors that could interfere with the outcomes of our study.

Seventy-seven percent of the total sample were women and 23% were men (*χ*2 = 162.84, *p* < 0.001). The mean age of the participants was 38.52 (± SD = 15.23) with an age range of 18–84 years. All participants belonged to medium to low socioeconomic segments of the Rio de Janeiro population and no students from the university itself were included. The SAP at PUC-Rio has been providing psychological assistance services at a symbolic price to individuals of low socioeconomic backgrounds of Rio de Janeiro for years (Féres-Carneiro, 2013). The SAP is responsible for the professional training of undergraduate students from the Department of Psychology, which is a mandatory requirement for obtaining a degree in psychology at PUC-Rio. Another major goal of the SPA is to provide psychological assistance to low-income populations in the vicinity of PUC-Rio. It offers services for all age groups, from children to adults, including families. Additionally, it provides neuropsychological assessment and treatment for patients with addictive disorders, as well as those in need of psychiatric care. Demographic data is summarized in Table 1.

**Table 1:** Demographic data of individuals that sought psychological help before and during the COVID-19 pandemic

	Before		During	
	*N*	%	*N*	%
Sex				
Men	55	21%	70	24%
Women	204	79%	220	76%
Total	259	47%	290	53%
Age				
Missing data	0	0.00%	14	4.83%
18–24	54	20.85%	66	22.76%
25–34	61	23.55%	77	26.55%
35–44	40	15.44%	59	20.34%
45–64	85	32.82%	64	22.07%
65 or more	19	7.34%	10	3.45%
Education				
Missing data	2	0.77%	9	3.10%
Incomplete middle school	16	6.18%	11	3.79%
Completed middle school	14	5.41%	6	2.07%
Incomplete high school	6	2.32%	11	3.79%
Completed high school	88	33.98%	96	33.10%
Incomplete college education	56	21.62%	70	24.14%
College degree	77	29.73%	87	30.00%

### Data collection

Data was obtained from patient’s reports collected during in-person or remote interviews on the first-time individuals visited the SAP at PUC-Rio seeking psychological help. Interviews and filling out of report forms were conducted by undergraduate students in their senior year of the Psychology program at PUC-Rio. All reports were then approved by a supervisor. SPA’s report forms were standardized and consisted of five main parts: demographic information, clarification of the demand, description of procedures, progress of therapeutic sessions, and a conclusion of the case. For the purposes of this study, only the demographic and clarification of the demand sections were used. To guarantee the anonymity of patients, all identifying information was removed from demographic and textual data.

Reports were categorized according to the year and divided into two groups: before (2019) and during the pandemic period (2020 and 2021). Text excerpts were transcribed verbatim in Portuguese using a word processing software for later textual corpus analysis. All entries were double checked for accuracy. Certain symbols, such as dashes, quotation marks, and indents, were removed or substituted to allow for software analysis. The typical length of the text excerpts was 39.55 words (± SD = 32.17; Md = 29).

To avoid subjective bias, we opted to not exclude residual-text information (RI) from the sampled texts. As RI we considered words such as: arrive as, because, bring, clinic, complaint, demand, help, main, patient, search, regarding, SPA, seek, service, so, therapy, to, treatment, and view (see Tables S1 and S2). These words were frequently used to compose sentences such as “The patient sought care at the SPA clinic regarding […]”; “Arrived at the SPA bringing as the main complaint […]”; “Patient sought the therapy service bringing as demand […]”; “The patient was pursuing treatment in view of […]”; and “The patient was searching for treatment because […].”. While results from the lexical analysis used in this study depict these words as relevant, due to their high frequency, they are also often used in other parts of the text excerpts. Nonetheless, they clearly do not explain patients` complaints or demands, and we decided to keep them in the analysis rather than removing them for transparency's sake. Only clusters with 0% of RI were analyzed in terms of number, clusters containing RI were analyzed only in terms of the nature of the demand.

### Data analysis

All text excerpts were carefully read by two independent researchers to check for errors and inconsistencies prior to analysis. Next, a lexical analysis of text excerpts was performed using Iramuteq (v.0.7) and R (v.4.1.3; R Core Team, 2022; Bienemann et al., 2020; Ratinaud & Marchand, 2012). Descending Hierarchical Analysis (DHA) and Correspondence Factor Analysis (CFA) were conducted as previously described (Bienemann et al., 2020). Briefly, DHA was carried out to categorize the words in the corpus into distinct groups based on their co-occurrence and distribution patterns. The goal was to identify text clusters with specific meanings, relying on the similarity, association, and frequency of the words (Ratinaud & Marchand, 2012; Salviati, 2018). Selected lexical sets were then ranked by respective chi-square values and frequency (Idoiaga Mondragon et al., 2021). The inclusion of both words and categories in their respective clusters was based on the following criteria (Bienemann et al., 2020; Ratinaud & Marchand, 2012; Salviati, 2018): (i) a frequency greater than the mean of occurrences in the corpus, (ii) the word appears primarily in that class, with a frequency of 50% or more, and (iii) a chi-square value greater than 3.84 within the respective cluster, with significance level at *p* < 0.05 when df = 1 (Bienemann et al., 2020; Idoiaga Mondragon et al., 2021). The active forms selected for analysis were adjectives, adverbs, nouns, and verbs recognized by the Iramuteq dictionary as previously described (Idoiaga Mondragon et al., 2021; de Souza and Bussolotti, 2021). Next, based on the DHC analysis, CFA analyses were carried out allowing for the visualization of relationships between lexical groups and descriptive variables in a factorial plane (Bienemann et al., 2020). Specifically, using the chi-squared (*χ*2) correlation and frequency values of each word in the corpus this analysis illustrates the proximities, oppositions, and tendencies of text segments (TS) in Cartesian space (Bienemann et al., 2020; Ratinaud & Marchand, 2012; Salviati, 2018; de Souza and Bussolotti, 2021).

### Ethical issues

Everyone who seeks treatment at the SAP of PUC-Rio is asked to sign an informed consent allowing the use of their data to be used for scientific purposes. Patient information was anonymized and stored in a password-encrypted database. This study was reviewed and approved by the National Committee on Research Ethics–CONEP (CAAE# 60447722.6.0000.5282).

## Results

### Pre-pandemic period

**Fig. 1:**
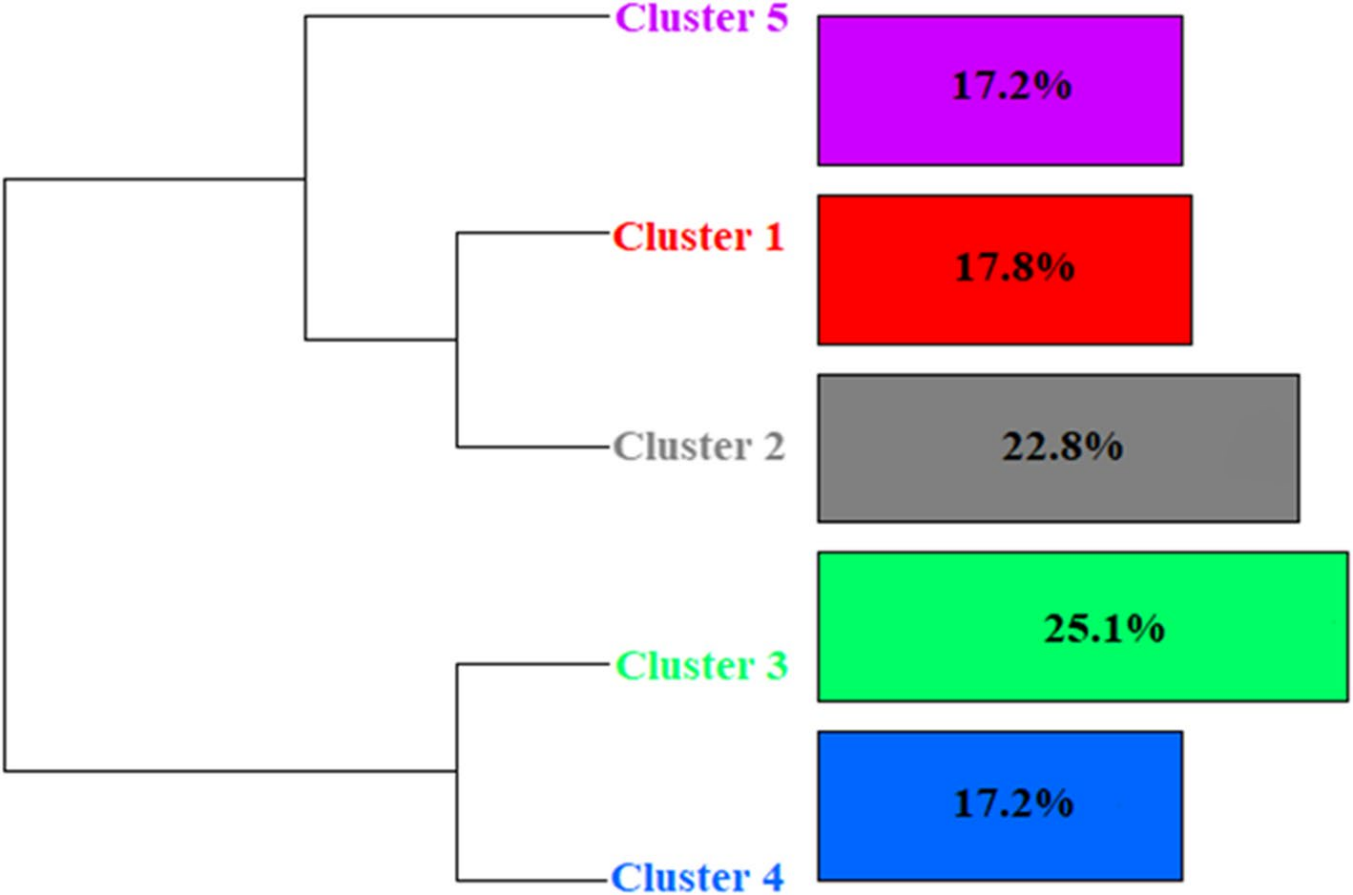
Dendrogram of the DHA for the pre-pandemic period

The DHA analysis resulted in 98.08% of the entire corpus being kept, which is considered an acceptable proportion for this type of analysis (2022; Bienemann et al., 2020; Ratinaud & Marchand, 2012). The corpus was divided into 260 text segments (TS), with 255 (98.08%) being saved. They contained 1843 words that appeared 9,489 times, with an average occurrence per TS of 36.49 and a standard deviation of 33.13, and a median of 25. Out of these, the active forms accounted for 1222 words, and 394 of these words had a frequency of more than three. Figure 1 illustrates the dendrogram of the DHA with 5 clusters for the pre-pandemic period. The analysis resulted in five clusters of words (Fig. 1 and Table 2): cluster 1 (red, 17.8% of classified text), cluster 2 (gray, 22.8%), cluster 3 (green, 25.1%), cluster 4 (blue, 17.2%), and cluster 5 (purple, 17.2%).

**Table 2:**
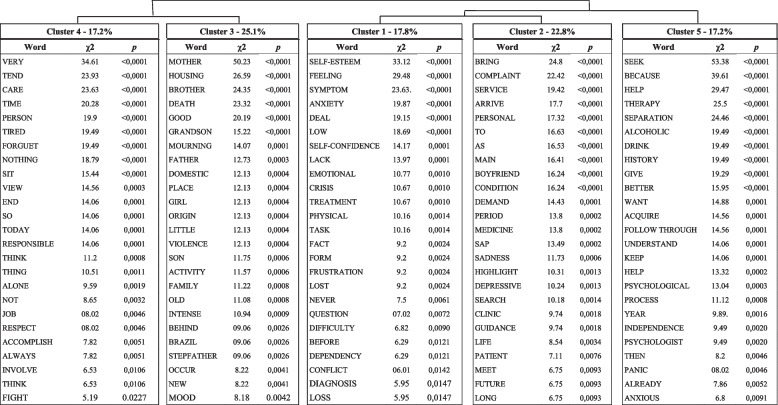
Dendrogram for the pre-pandemic period with the 25 words with highest χ2 in each cluster

Cluster 1 (red) corresponds to self-esteem factors. Forms significantly associated with cluster 1 were: self-esteem (< 0.0001), feeling (< 0.0001), symptom (< 0.0001), anxiety (< 0.0001), coping (< 0.0001), low (< 0.0001), and self-confidence (0.00016). Self-esteem factors appears to be closed related to (a) anxiety, “Work on anxiety disorder, paranoid health-related thoughts, lack of self-esteem, insecurity, self-validation, thoughts that the worst will always happen.”; (b) depression, “Frequent feeling of emptiness, in addition to issues related to low self-esteem associated with a feeling of inferiority compared to people in their social cycle,” “Doesn't like to leave the house, can't go out alone and doesn't like to dress up because she doesn't feel pretty, she complains of low self-esteem. In view of all these related issues she no longer has the will to live.”; and (c) work life, “After being fired from a job she loved, in 2017, she went through a period of great discouragement, anxiety and negative thoughts about the future and herself. She spent about 2 years without doing the activities she liked, gained weight and until then had not been able to regain her self-esteem and her identity.”

Cluster 2 (gray) corresponds to mental health. The forms significantly with cluster 2 were bring (< 0.0001), complaint (< 0.0001), service (< 0.0001), condition (< 0.0001), medicine (0.0002), SAP (0.0002), sadness (0.0006), depressive (0.0013), and anxiety (0.0109). Despite containing a lot of residual information (RI, see “Material and methods” section and Table S1), this cluster represents mostly disorders and medication related issues such as (a) medication effectiveness, “[…] claimed that she felt that the medication was no longer controlling her anxiety.”; (b) dissatisfaction with medication, “[…] wants to stop taking antidepressant medication.”; (c) subjective relations to medication, “She arrives at the service under the guidance of her boyfriend, coming from psychiatric care. She highlights her anxiety, her relationship with alcohol, and her current use of medication.”; (d) identification of medication, “Reports having had moments of extreme sadness in which she spent days without leaving the house, brushing her teeth and taking a shower, even thinking about suicide. The patient currently uses Luvox (fluvoxamine) and is assisted by a psychiatrist.” In addition to medication and disorders issues, we can also find plural and isolated demands, such as strong feelings of sadness or anxiety, “The patient arrived at the SAP with anxiety as her main complaint, […] bothered by a state of irritation that she considers excessive, explosive temper, and feelings of guilt.”

Cluster 3 (green) corresponds to family dynamics. Forms significantly associated with cluster 3 were: mother (< 0.0001), housing (< 0.0001), brother (< 0.0001), death (< 0.0001), good (< 0.0001), grandson (< 0.0001), mourning (0.0001), father (0.0003), violence (0.0004), child (0.0006), and family (0.0008). In this cluster, it is possible to identify aspects related to the composition and degree of kinship of the members of the family group. Particularly, the relationships between them, their level of integration or dissociation, proximity or separation, relationship problems, boundaries, and other family related interactions. Among these we can mention text excerpts such as “He currently lives with his mother, stepfather and brother, and has a good relationship with everyone. She never had contact with her father.”, “She has a family history of mental illness (father, mother and sister) and domestic violence, she was beaten by her father in adolescence.”, and “She is the eldest daughter coming from a family with limited financial resources. Her father and mother treat her like a sister, putting her as the family's caretaker.”

Cluster 4 (blue) corresponds to loneliness, tiredness and exhaustion. Forms significantly associated with cluster 4 were: very (< 0.0001), tend (< 0.0001), care (< 0.0001), time (< 0.0001), tired (< 0.0001), forget (< 0.0001), responsible (0.0001), alone (0.0019), and employment (0.0046). This cluster seems to be associated with multifactorial demands: “She reports that she is very tired, she is responsible for her 3 children in addition to attending college.”, “[…] she was unable to do anything at work because she forgets things. According to her, she feels very tired and cannot stop at any job because she feels uncomfortable around other people.”, “[…] ended up pushing everyone away from her life and today she feels very alone, with no one to share anything with, and says she is responsible for that due to her arrogance.”, and “[…] reports that she invests practically all of her time in favor of her family and ends up not having time for herself.”

Cluster 5 (purple) corresponds to RI (see “Material and methods” section) and indicators of motivation to seek therapy. Forms significantly linked to cluster 5 were seek (< 0.0001), because (< 0.0001), help (< 0.0001), therapy (< 0.0001), separation (< 0.0001), history (< 0.0001), drink (< 0.0001), alcoholic (< 0.0001), better (< 0.0001), want (0.0001), follow through (0.0001), acquire (0.0001), keep (0.0001), understand (0.0001), and help (0.0002). In this cluster we have a set of multifactorial demands associated with RI, as noted in the following excerpt: “sought psychotherapy as a means of trying to better understand her issue."

### During the pandemic period

**Fig. 2:**
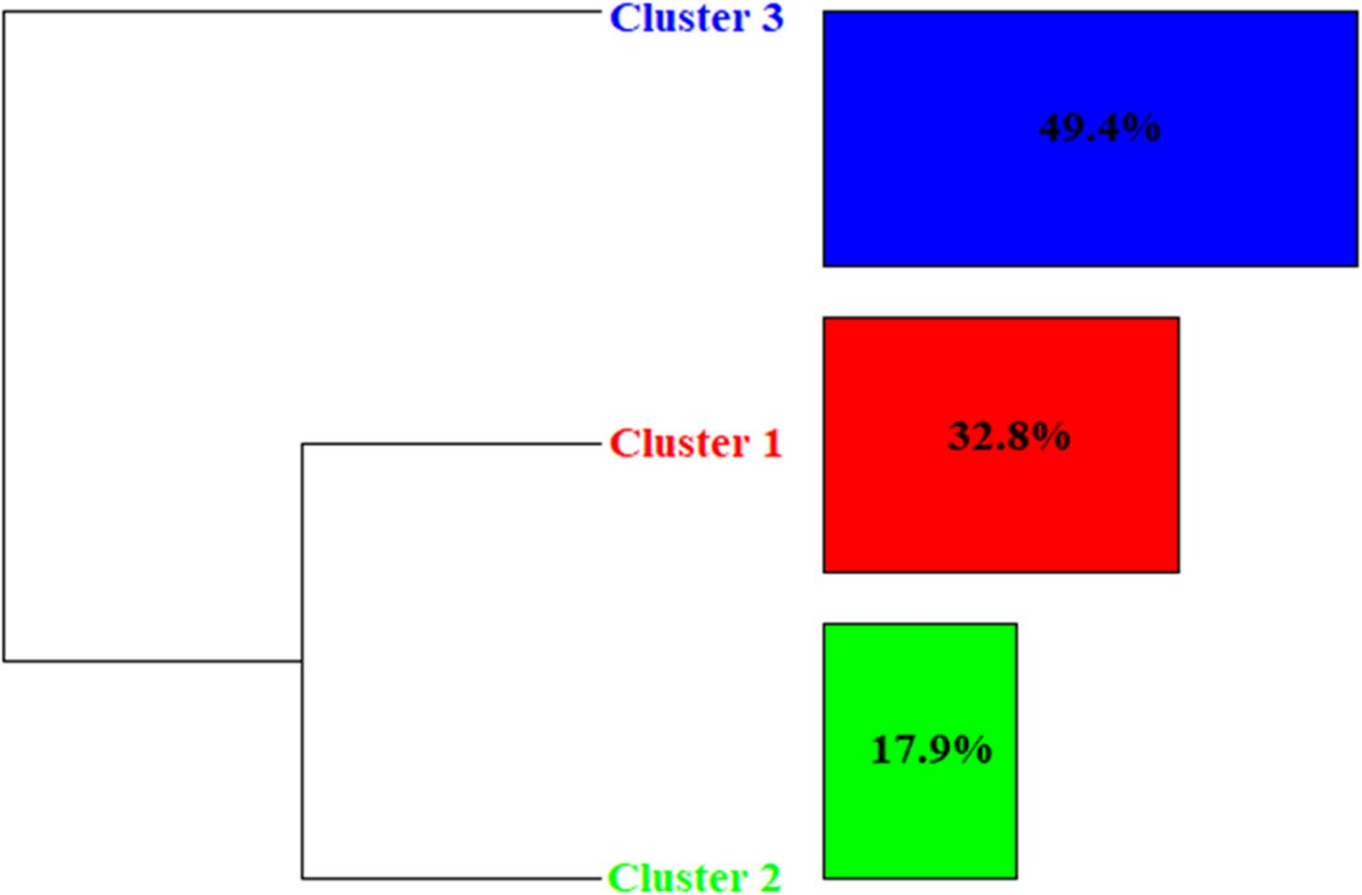
Dendrogram of the DHA during the pandemic period

The corpus was divided into 279 TS, of which 235 (84,23%) were retained. The number of words was 1,987 that occurred 10,212 times (mean of occurrence for TS = 36.60). In addition, 1,336 active words were identified, with 434 words with a frequency greater than three. The DHA analysis resulted in three clusters (Fig. 2 and Table 3). The clustering resulted in only two branches, with cluster 1 (in red, representing 32.8% of the classified text) and cluster 2 (in green, 17.9%) in one branch, and cluster 3 (in blue, 49.4%) in the other.

**Table 3:**
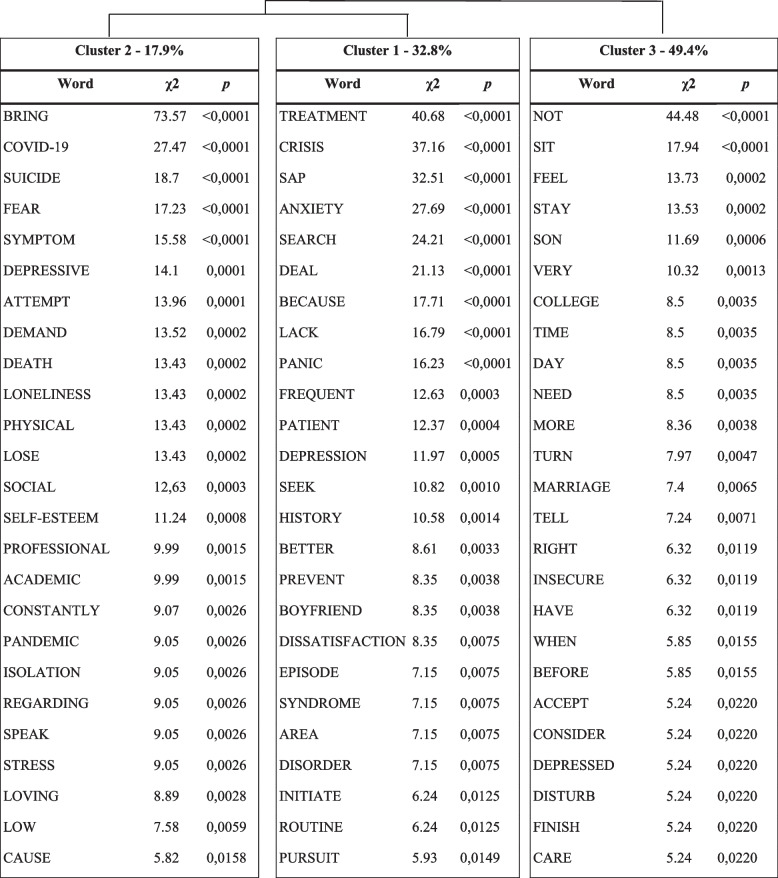
Dendrogram for the during the pandemic period with the 25 words with highest *χ*2 in each cluster.

Cluster 1 (red) corresponds to anxiety symptoms and panic attacks. The forms significantly associated with cluster 1 were: treatment (< 0.0001), crisis (< 0.0001), SAP (< 0.0001), anxiety (< 0.0001), coping (< 0.0001), panic (< 0.0001), depression (0.0005), and disorder (0.0075). In this cluster complaints seem mostly related to anxiety and panic attacks: “The patient has complaints related to unwillingness to do your routine activities and frequent anxiety attacks”, “Sought care due to stressful relationships with family and history of panic attacks at work and at home”, “Patient seeks care due to severe anxiety attacks”, and “The patient reported having sought therapeutic care for having panic and anxiety attacks, complaining of difficulties in dealing with anxiety and symptoms of panic disorder.”

Cluster 2 (green) corresponds to grief and mental illness related to the COVID-19 pandemic. The forms significantly related to cluster 2 were COVID-19 (< 0.0001), suicide (< 0.0001), fear (< 0.0001), symptom (< 0.0001), depressive (< 0.0001), loss (0.0002), loneliness (0.0002), death (0.0002), self-esteem (0.0003), social (0.0003), professional (0.0008), pandemic (0.0026), and stress (0.0026). As noted in the following excerpts: “[…] grief from close friends and psychosomatic symptoms of stress, anxiety and fear related to the COVID-19 pandemic […] the patient described a lot of sadness after the death of his grandmother and sought care after a recommendation from a friend”, “Sought care for anxiety and depressive symptoms that he developed after having COVID-19 and losing his father in July”, “Patient brings grief issues and psychosomatic symptoms of stress, anxiety and fear related to the COVID-19 pandemic”, and “[…] recurring doubts about his academic and professional trajectory, social isolation, anxiety and grief.”

Cluster 3 (blue) corresponds to conflicts and communication between family members. Forms significantly associated with cluster 3 were not (< 0.0001), feel (0.0002), child (0.0006), college (0.0035), time (0.0035), need (0.0035), and marriage (0.0065). As someone can see in these examples: “Constantly questions his place in family groups and among friends, does not consider himself as being an official member and feels like he can be removed if he end up bothering others […] he feels the need to take care of others even if it brings harm to him and is not comfortable taking care of himself”, “Her main complaint was that she really wanted to have a relationship with her son, but she couldn't because of her husband, who was very controlling.”, “Legal fight with ex-wife to obtain shared custody of daughter”, “Feels insecure and frustrated because he believes he is not being able to satisfy his current partner sexually”, “considers herself a very closed person who keeps all her feelings inside, she does not know how to say no to her family, and says she feels overwhelmed all the time because of this.”

### Correspondence factor analysis (CFA)

#### Pre-pandemic period

**Fig. 3:**
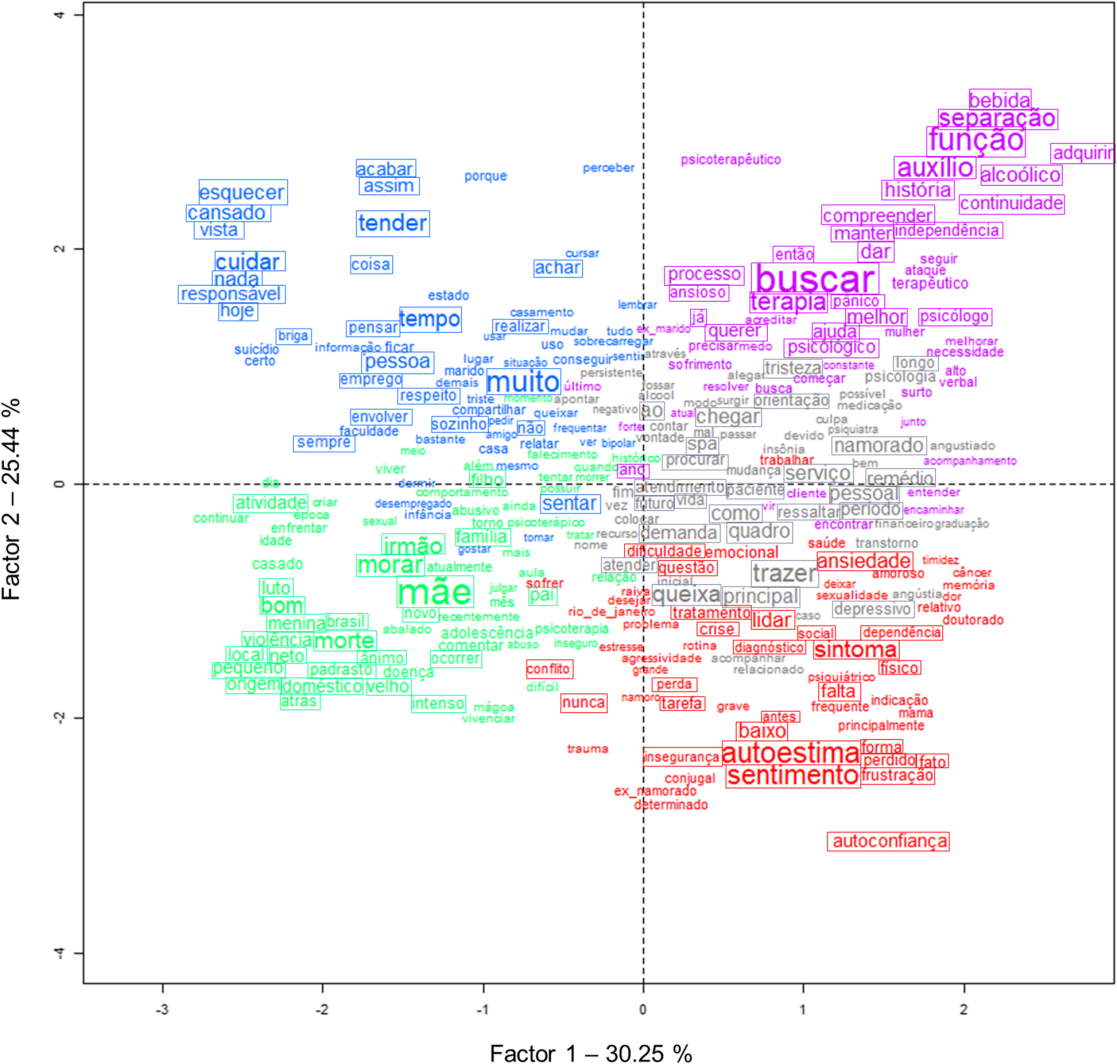
Correspondence factorial analysis (CFA) of the pre-pandemic period. Words in red belong to Cluster 1 that relates to self-esteem factors, words in gray belong to Cluster 2 that relates to mental health, words in green belong to Cluster 3 that relates to family dynamics, words in blue belong to Cluster 4 that relates to loneliness, tiredness and exhaustion, and words in purple belong to Cluster 5 that relates to RI and indicators of motivation to seek therapy. Highlighted words by solid rectangles represent the 25 words with highest χ2 in each cluster for text excerpts collected during the pre-pandemic period.

CFA was carried out to visualize the relationship between clusters (Bienemann et al., 2020; Ratinaud & Marchand, 2012; Salviati, 2018). While five clusters were identified, the boundaries between them overlap (Fig. 3). As mentioned above, words in red belong to Cluster 1 that relates to self-esteem factors, words in gray belong to Cluster 2 that relates to mental health, words in green belong to Cluster 3 that relates to family dynamics, words in blue belong to Cluster 4 that relates to loneliness, tiredness and exhaustion, and words in purple belong to Cluster 5 that relates to RI (see “Material and methods” section and Table S1) and indicators of motivation to seek therapy. Highlighted words by solid rectangles represent the 25 words with highest χ2 in each cluster for text excerpts collected during the pre-pandemic period (Table 2). The two factors explain 30.58% (*X* axis) and 25.57% (*Y* axis) of the model, respectively (Fig. 3).

### During-pandemic period

**Fig. 4:**
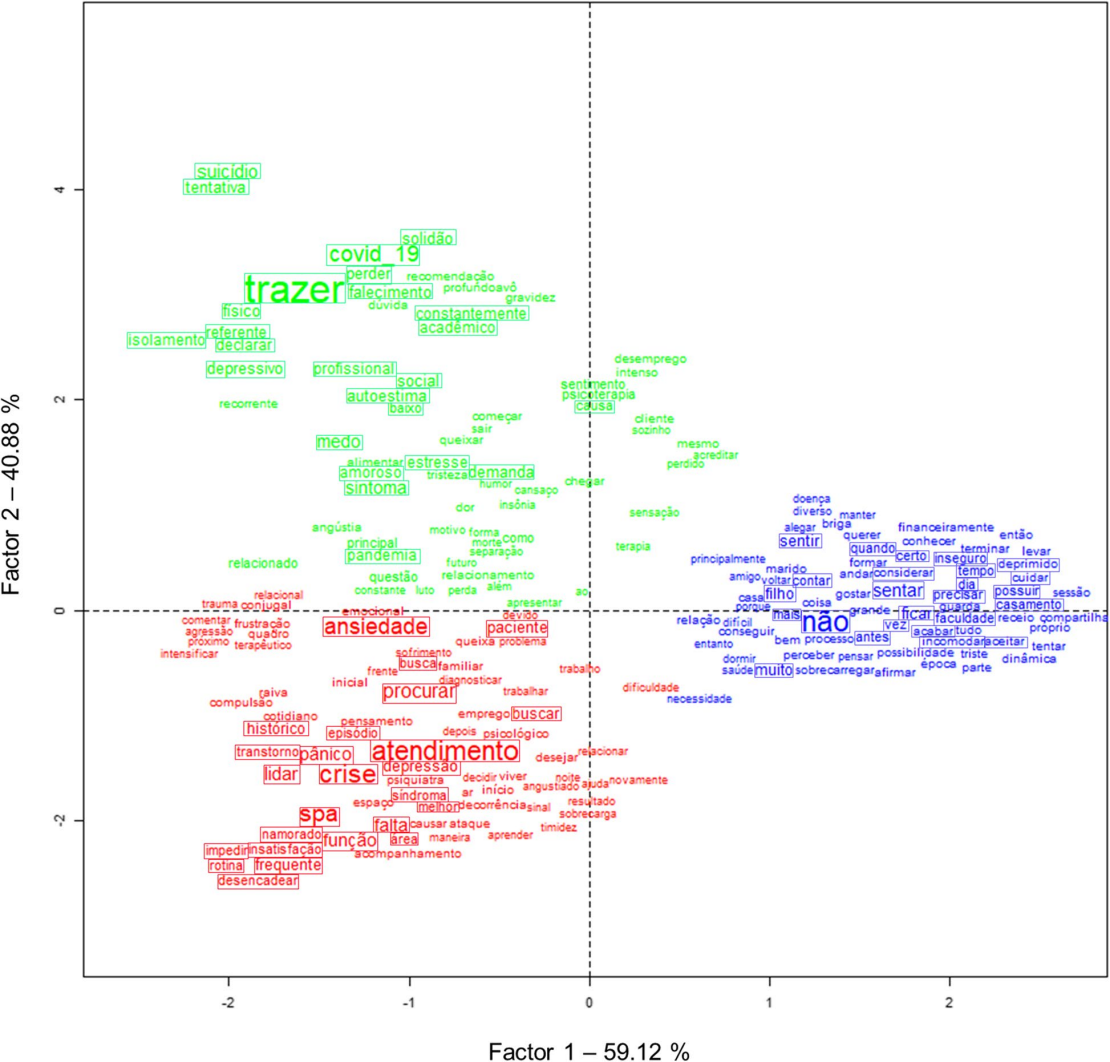
Correspondence factorial analysis (CFA) of the during-pandemic period. Words colored red are part of Cluster 1, which pertains to anxiety symptoms and panic attacks. Green words are associated with Cluster 2, which covers grief and mental health conditions related to the COVID-19 pandemic. Blue words belong to belong to Cluster 3, which relates to conflicts and communication among family members.

Contrary to the pre-pandemic period, identified clusters are well-defined showing clear boundaries (Fig. 4). As cited above words in red belong to Cluster 1 that relates to anxiety symptoms and panic attacks, words in green belong to Cluster 2 that relates to grief and mental illness related to the COVID-19 pandemic, and words in blue belong to Cluster 3 that relates to conflicts in family and relationships. Highlighted words by solid rectangles represent the 25 words with highest *χ*2 in each cluster for text excerpts collected during the pandemic period (Table 3). In this group, the two factors explain 58.39% (*X* axis) and 41.61% (*Y* axis) of the model, respectively which is represented on the *X* and *Y* axes in (Fig. 4).

Therefore, at the pre-pandemic moment, we can highlight the strong proximity between clusters 1 (self-esteem) and 2 (mental health), as demonstrated by the CFA (Fig. 3). The association between both suggests the implication of self-esteem factors on mental health disorders. Likewise, clusters 3 and 4 are also closely related (Fig. 3) and imply insights into conflicts and communication between family members and its correlation with the feeling of loneliness, tiredness, and exhaustion in this population even before the pandemic. Based on these analyses, we can begin to identify a few changes in the main demand and redirection of complaints of patients during the period of COVID-19.

## Discussion

This is a mixed methods study in the sense that made use of a quanti-qualitative analysis to identify and compare the main complains and demands from individuals seeking psychological help before the onset (2019) and during the COVID-19 pandemic (2020 and 2021), with particular interest in those living in low-income communities. By now, the impact of the pandemic on mental health is evidently clear, as evidenced by the more than 35,000 papers published on this topic (Penninx et al., 2022). However, most of studies do not provide a complete picture of such effect due, for instance, pre-COVID-19 data (Penninx et al., 2022). Therefore, despite its inherent limitations, the study of relevant mental health data from patient records using the current approach is a straightforward and low-cost way to investigate clinical changes over time. Moreover, like observed in the present study, patient report-forms have valuable demographic information, which, for instance, allows carrying analyses based on secondary speech that guarantees methodological support and reliability (Ahmed, 1996). By “secondary speech” we mean that our data was not directly extracted from transcripts of patients’ verbal responses but rather from the notes taken by the students who conducted the interviews. Of course, for this to be possible it is worth reminding that all ethical requirements for conducting such studies are met, including ethical research requirements of institutions. To begin with, the fact that most of the demands are made by women, both before and during the pandemic, may indicate a stigma behind men from lower social classes seeking psychological support. Interestingly, according to our data, the pandemic seems to have had no influence on this scenario. The frequency of demands from both sexes remained similar for both periods before and during the pandemic (Table 1). In fact, previous studies indicate that in Brazil the stigma associated with mental illness is still a sad reality (Weber & Juruena, 2017) and, as one could expect, manifests itself more sharply in more vulnerable and low-income communities (Kohn et al., 2018), a pattern that is also observed in other developing countries (Ganasen et al., 2008). Yet, one silver lining amid all the pain, hardship, and suffering brought by the COVID-19 pandemic, might be an increased awareness and understanding of mental health issues. In fact, Brazil is taking important steps to expand access to mental health treatment and reduce the stigma on mental health, particularly among the less privileged (Carvalho et al., 2021; de Oliveira et al., 2022). In this regard, it is worth mentioning the pioneering work of the SAP at PUC-Rio, which has been providing psychological assistance to those in need since 1957 (Féres-Carneiro, 2013).

Our study shows a clear change in the pattern and specificity of psychological issues and demands during the pandemic period. We believe that our data revealed that this change is related either to the number or, on certain occasions, to the nature of complaints. For instance, note that clusters associated with family dynamics (cluster 3 on both periods) not only remained, but also increased by 24% during the pandemic. In both periods, complaints of this nature proved to be the main reason for seeking psychological treatment at SAP. It is important to identify long-lasting consequences from changes in family dynamics and perceptions once it might disrupt the bond, connection, affection, and quality of the family relationship.

Furthermore, the identification of a specific cluster for symptoms of anxiety and panic attacks during the pandemic period raises concerns about the worsening of problems related to mental health. Fear of contamination itself in addition to forced quarantine and lockdowns can produce acute panic and anxiety in the long run (Dubey et al., 2020). Given the fear and uncertainty experienced by the general population due to the pandemic, it is reasonable to assume that there would be an associated increase and/or intensification of anxiety and panic disorder symptoms. Indeed, evidence shows that getting infected with the virus or witnessing loved ones experience COVID-19 symptoms can trigger anxiety symptoms and/or panic attacks (Javelot & Weiner, 2021).

In addition, our study highlighted one cluster specific for grief and mental illness related to the COVID-19 pandemic. The losses and grief felt during this period of crisis constitute risk factors for the development of psychological disorders, even to a greater degree when these feelings are not properly supported. Loss and grief identification and management among patients, family members, and healthcare professionals are critically important during the COVID-19 pandemic (Tao et al., 2022). Studies indicate that current operating guidelines have proven insufficient in managing loss and grief, calling attention to the need for new strategies to tackle the many dimensions of such feelings (Tao et al., 2022).

With regard to the CFA analyses, we can observe that in the period before the pandemic, complaints of different clusters are mainly overlapping, especially between cluster 1 (self-esteem) and 2 (mental health and medication). This may indicate a correlation between factors related to low self-esteem and the development of psychological disorders. Likewise, clusters 3 and 4 are also closely related (Fig. 3) and imply insights into family dynamics and its correlation with the feeling of loneliness, tiredness, and exhaustion in this population even before the pandemic and quarantine measures.

In contrast, during the pandemic, the CFA analysis showed better-defined and less overlapping clusters in addition to a smaller number of them. When the patient brings only one complaint, it does not necessarily mean a better situation than the patient who brings plural complaints, but this may indicate a better-defined perception of the main complaint. If before the pandemic patients reported a plurality of complaints, during the pandemic there was a greater focus on a given subject. This can designate a perception of prominence or aggravation of a specific problem, such as (1) anxiety symptoms and panic attacks, (2) grief and mental illness related to the COVID-19 pandemic, and / or (3) conflicts and communication between family members.

## Conclusion

In conclusion, the COVID-19 pandemic has had a devastating impact on low-income communities, exacerbating existing symptoms, social-economic inequalities, and making it more difficult for these communities to access adequate healthcare and support services (Bassey et al., 2022; Pereira & Oliveira, 2020; Rohatgi & Dash, 2023). To address these challenges, it is crucial that governments and organizations provide targeted support to mitigate the profound and long-lasting effects of the pandemic on mental health. Moreover, it is important to raise awareness about the subjective ways in which the pandemic affected emotionally, and to destigmatize seeking help. Although extremely necessary for the time, restrictive measures such as social isolation, quarantine and lock down had major effects on mental health. In our study, the data allowed us to better understand some of the psychological demands during the COVID-19 pandemic. This is showcased by the review and analyses of the reports, as well by the interface with the literature. To a lesser degree, our results might help to have a better design when planning the community mental health services.

The study has an important limitation, concerning the sample, the prior elaboration of the reports and methodology. It is important to note that our sample is predominantly composed of women (77.23%). Therefore, our results most likely reflect psychological issues within a women’s framework. However, at this point, we cannot directly compare our findings to previous studies showing that women tend to seek mental health services more than men (Liddon et al., 2018; Reily et al., 2023; Seidler et al., 2016). With regard to the reports, as a specific interview was not carried out with the patients, a considerable amount of residual information from textual construction and contextualization interfered with the results. We are also aware that the lexical interpretation and associations within a particular cluster rely on the inherent subjectivity of the person conducting the study (Góes et al., 2021; Monteiro et al., 2021; Lima et al., 2022). The exploratory and qualitative methodologies of the study provide different interpretations; however, as the data from this study guarantees anonymity and cannot be nominally published, we provide as much information as possible. Nevertheless, this study offers a first finding about possible complaint transitions due to the pandemic. While the results from this study are open to distinct interpretations, they demonstrate changes in the reasons why people sought psychological help during the pandemic. Our findings contribute to the current literature, highlighting the impact of COVID-19 on mental health (Penninx et al., 2022), by illustrating changes in lexical patterns in psychological reports from individuals with low socioeconomic backgrounds living in Rio de Janeiro, Brazil. Whether or not our observations can be directly linked to specific individual and situational factors that contribute to negative mental health outcomes following the pandemic remains elusive. Long-term health problems may arise over the years and following up on the evolution of demands in health care institutions is an important indicator of what we should be aware of when dealing with economically vulnerable populations. Experts already predict that mental health consequences of the COVID-19 crisis are likely to be present for a longer period of time and may also peak later than the current pandemic (Sher, 2020). In that way, understanding the development of psychological symptoms in a vulnerable population has become paramount after the COVID-19 pandemic. These results can be useful for better understanding the effects of the COVID-19 pandemic on mental health beyond tracking its long-lasting consequences. Beyond that, we hope to help professionals and care services in the management of these issues, in addition to shedding light on risk factors for the development of psychological disorders.

### Supplementary Information

**Additional file 1: Table S1.** Translation and classification of words with the 25 words with highest χ^2^ in each cluster of the pre-pandemic group. **Table S2.** Translation and classification of words with the 25 words with highest χ^2^ in each cluster of the during-pandemic group.

## Data Availability

The data used in this study is not available online, and we cannot share it as it is restricted by ethical issues. The files contain confidential information and require anonymity, preventing the identification of participants.

## Abbreviations

SAP: Service of Applied Psychology
PUC-Rio: Pontifical Catholic University of Rio de Janeiro
RI: Residual information
DHA: Descending Hierarchical Analysis
CFA: Correspondence Factor Analysis
TS: Text segment
