# Supplementary material for: Tracking demands for seeking psychological help before and during the COVID-19 pandemic: a quanti-qualitative study
Source: Psicol Reflex Crit. 2023 Aug 29;36:22. doi: 10.1186/s41155-023-00264-0 (PMC10462552; doi:10.1186/s41155-023-00264-0)
Supplement: Supplementary file 1 — Additional file 1: Table S1. Translation and classification of words with the 25 words with highest χ2 in each cluster of the pre-pandemic group. Table S2. Translation and classification of words with the 25 words with highest χ2 in each cluster of the during-pandemic group. [file 41155_2023_264_MOESM1_ESM.docx]

**Table S1.** Translation and classification of words with the 25 words with highest χ^2^ in each cluster of the pre-pandemic group.

| **Cluster 4 - 17.2%** | | |  | **Cluster 3 - 25.1%** | | |  | **Cluster 1 - 17.8%** | | |  | **Cluster 2 - 22.8%** | | |  | **Cluster 5 - 17.2%** | | |
| --- | --- | --- | --- | --- | --- | --- | --- | --- | --- | --- | --- | --- | --- | --- | --- | --- | --- | --- |
| **WE** | **WP** | **WC** |  | **WE** | **WP** | **WC** |  | **WE** | **WP** | **WC** |  | **WE** | **WP** | **WC** |  | **WE** | **WP** | **WC** |
| VERY | MUITO | MW |  | MOTHER | MÃE | MW |  | SELF-ESTEEM | AUTOESTIMA | MW |  | BRING | TRAZER | RI |  | SEEK | BUSCAR | RI |
| TEND | TENDER | MW |  | HOUSING | MORAR | MW |  | FEELING | SENTIMENTO | MW |  | COMPLAINT | QUEIXA | RI |  | BECAUSE | FUNÇÃO | RI |
| CARE | CUIDAR | MW |  | BROTHER | IRMÃO | MW |  | SYMPTOM | SINTOMA | MW |  | SERVICE | SERVIÇO | RI |  | HELP | AUXÍLIO | RI |
| TIME | TEMPO | MW |  | DEATH | MORTE | MW |  | ANXIETY | ANSIEDADE | MW |  | ARRIVE | CHEGAR | RI |  | THERAPY | TERAPIA | RI |
| PERSON | PESSOA | MW |  | GOOD | BOM | MW |  | DEAL | LIDAR | MW |  | PERSONAL | PESSOAL | MW |  | SEPARATION | SEPARAÇÃO | MW |
| TIRED | CANSADO | MW |  | GRANDSON | NETO | MW |  | LOW | BAIXO | MW |  | TO | AO | RI |  | ALCOHOLIC | ALCOÓLICO | MW |
| FORGUET | ESQUECER | MW |  | MOURNING | LUTO | MW |  | SELF-CONFIDENCE | AUTOCONFIANÇA | MW |  | AS | COMO | RI |  | DRINK | BEBIDA | MW |
| NOTHING | NADA | MW |  | FATHER | PAI | MW |  | LACK | FALTA | MW |  | MAIN | PRINCIPAL | RI |  | HISTORY | HISTÓRIA | MW |
| SIT | SENTAR | MW |  | DOMESTIC | DOMÉSTICO | MW |  | EMOTIONAL | EMOCIONAL | MW |  | BOYFRIEND | NAMORADO | MW |  | GIVE | DAR | MW |
| VIEW | VISTA | RI |  | PLACE | LOCAL | MW |  | CRISIS | CRISE | MW |  | CONDITION | QUADRO | MW |  | BETTER | MELHOR | MW |
| END | ACABAR | MW |  | GIRL | MENINA | MW |  | TREATMENT | TRATAMENTO | MW |  | DEMAND | DEMANDA | RI |  | WANT | QUERER | MW |
| SO | ASSIM | RI |  | ORIGIN | ORIGEM | MW |  | PHYSICAL | FÍSICO | MW |  | PERIOD | PERÍODO | MW |  | ACQUIRE | ADQUIRIR | MW |
| TODAY | HOJE | MW |  | LITTLE | PEQUENO | MW |  | TASK | TAREFA | MW |  | MEDICINE | REMÉDIO | MW |  | FOLLOW THROUGH | CONTINUIDADE | MW |
| RESPONSIBLE | RESPONSÁVEL | MW |  | VIOLENCE | VIOLÊNCIA | MW |  | FACT | FATO | MW |  | SAP | SPA | RI |  | UNDERSTAND | COMPREENDER | MW |
| THINK | ACHAR | MW |  | SON | FILHO | MW |  | FORM | FORMA | MW |  | SADNESS | TRISTEZA | MW |  | KEEP | MANTER | MW |
| THING | COISA | MW |  | ACTIVITY | ATIVIDADE | MW |  | FRUSTRATION | FRUSTRAÇÃO | MW |  | HIGHLIGHT | RESSALTAR | MW |  | HELP | AJUDA | MW |
| ALONE | SOZINHO | MW |  | FAMILY | FAMÍLIA | MW |  | LOST | PERDIDO | MW |  | DEPRESSIVE | DEPRESSIVO | MW |  | PSYCHOLOGICAL | PSICOLÓGICO | MW |
| NOT | NÃO | MW |  | OLD | VELHO | MW |  | NEVER | NUNCA | MW |  | SEARCH | PROCURAR | RI |  | PROCESS | PROCESSO | MW |
| JOB | EMPREGO | MW |  | INTENSE | INTENSO | MW |  | QUESTION | QUESTÃO | MW |  | CLINIC | ATENDIMENTO | RI |  | YEAR | ANO | MW |
| RESPECT | RESPEITO | MW |  | BEHIND | ATRÁS | MW |  | DIFFICULTY | DIFICULDADE | MW |  | GUIDANCE | ORIENTAÇÃO | MW |  | INDEPENDENCE | INDEPENDÊNCIA | MW |
| ACCOMPLISH | REALIZAR | MW |  | BRAZIL | BRASIL | MW |  | BEFORE | ANTES | MW |  | LIFE | VIDA | MW |  | PSYCHOLOGIST | PSICÓLOGO | MW |
| ALWAYS | SEMPRE | MW |  | STEPFATHER | PADRASTO | MW |  | DEPENDENCY | DEPENDÊNCIA | MW |  | PATIENT | PACIENTE | RI |  | THEN | ENTÃO | MW |
| INVOLVE | ENVOLVER | MW |  | OCCUR | OCORRER | MW |  | CONFLICT | CONFLITO | MW |  | MEET | ATENDER | MW |  | PANIC | PÂNICO | MW |
| THINK | PENSAR | MW |  | NEW | NOVO | MW |  | DIAGNOSIS | DIAGNÓSTICO | MW |  | FUTURE | FUTURO | MW |  | ALREADY | JÁ | MW |
| FIGHT | BRIGA | MW |  | MOOD | ÂNIMO | MW |  | LOSS | PERDA | MW |  | LONG | LONGO | MW |  | ANXIOUS | ANSIOSO | MW |
| RI = 8% | | |  | RI = 0% | | |  | RI = 0% | | |  | RI = 48% | | |  | RI = 16% | | |
| WE, word in English; WP, word in Portuguese; MW, meaningful word; WC, word classification; %RI = frequency of RIs within the 25 words with highest χ^2^ | | | | | | | | | | | | | | | | | | |

**Table S2.** Translation and classification of words with the 25 words with highest χ^2^ in each cluster of the during-pandemic group.

| **Cluster 2 - 17.9%** | | | |  | **Cluster 1 - 32.8%** | | |  | **Cluster 3 - 49.4%** | | |
| --- | --- | --- | --- | --- | --- | --- | --- | --- | --- | --- | --- |
| **WE** | | **WP** | **C** |  | **WE** | **WP** | **C** |  | **WE** | **WP** | **C** |
| BRING | | TRAZER | RI |  | TREATMENT | ATENDIMENTO | RI |  | NOT | NÃO | MW |
| COVID-19 | | COVID-19 | MW |  | CRISIS | CRISE | MW |  | SIT | SENTAR | MW |
| SUICIDE | | SUICÍDIO | MW |  | SAP | SPA | RI |  | FEEL | SENTIR | MW |
| FEAR | | MEDO | MW |  | ANXIETY | ANSIEDADE | MW |  | STAY | FICAR | MW |
| SYMPTOM | | SINTOMA | MW |  | SEARCH | PROCURAR | RI |  | SON | FILHO | MW |
| DEPRESSIVE | | DEPRESSIVO | MW |  | DEAL | LIDAR | MW |  | VERY | MUITO | MW |
| ATTEMPT | | TENTATIVA | MW |  | BECAUSE | FUNÇÃO | RI |  | COLLEGE | FACULDADE | MW |
| DEMAND | | DEMANDA | RI |  | LACK | FALTA | MW |  | TIME | TEMPO | MW |
| DEATH | | FALECIMENTO | MW |  | PANIC | PÂNICO | MW |  | DAY | DIA | MW |
| LONELINESS | | SOLIDÃO | MW |  | FREQUENT | FREQUENTE | MW |  | NEED | PRECISAR | MW |
| PHYSICAL | | FÍSICO | MW |  | PATIENT | PACIENTE | RI |  | MORE | MAIS | MW |
| LOSE | | PERDER | MW |  | DEPRESSION | DEPRESSÃO | MW |  | TURN | VEZ | MW |
| SOCIAL | | SOCIAL | MW |  | SEEK | BUSCAR | RI |  | MARRIAGE | CASAMENTO | MW |
| SELF-ESTEEM | | AUTOESTIMA | MW |  | HISTORY | HISTÓRICO | MW |  | TELL | CONTAR | MW |
| PROFESSIONAL | | PROFISSIONAL | MW |  | BETTER | MELHOR | MW |  | RIGHT | CERTO | MW |
| ACADEMIC | | ACADÊMICO | MW |  | PREVENT | IMPEDIR | MW |  | INSECURE | INSEGURO | MW |
| CONSTANTLY | | CONSTANTEMENTE | MW |  | BOYFRIEND | NAMORADO | MW |  | HAVE | POSSUIR | MW |
| PANDEMIC | | PANDEMIA | MW |  | DISSATISFACTION | INSATISFAÇÃO | MW |  | WHEN | QUANDO | MW |
| ISOLATION | | ISOLAMENTO | MW |  | EPISODE | EPISÓDIO | MW |  | BEFORE | ANTES | MW |
| REGARDING | | REFERENTE | RI |  | SYNDROME | SÍNDROMA | MW |  | ACCEPT | ACEITAR | MW |
| SPEAK | | DECLARAR | MW |  | AREA | ÁREA | MW |  | CONSIDER | CONSIDERAR | MW |
| STRESS | | ESTRESSE | MW |  | DISORDER | TRANSTORNO | MW |  | DEPRESSED | DEPRIMIDO | MW |
| LOW | | BAIXO | MW |  | INITIATE | DESENCADEAR | MW |  | DISTURB | INCOMODAR | MW |
| LOVING | | AMOROSO | MW |  | ROUTINE | ROTINA | MW |  | FINISH | ACABAR | MW |
| CAUSE | | CAUSA | MW |  | PURSUIT | BUSCA | RI |  | CARE | CUIDAR | MW |
| RI = 12% | | | |  | RI = 28% | | |  | RI = 0% | | |
| WE, word in English; WP, word in Portuguese; MW, meaningful word; WC, word classification; %RI = frequency of RIs within the 25 words with highest χ^2^ | | | | | | | | | | |  |
